# Association between value-based purchasing score and hospital characteristics

**DOI:** 10.1186/1472-6963-12-464

**Published:** 2012-12-17

**Authors:** Bijan J Borah, Michael G Rock, Douglas L Wood, Daniel L Roellinger, Matthew G Johnson, James M Naessens

**Affiliations:** 1 College of Medicine, Mayo Clinic, 200 First Street SW, Rochester, MN, 55905, USA; 2 Division of Health Care Policy & Research, Mayo Clinic, 200 First Street SW, Rochester, MN, 55905, USA; 3 Department of Orthopedic Surgery, Mayo College of Medicine, 200 First Street SW, Rochester, MN, 55905, USA; 4 Division of Cardiovascular Disease, Mayo College of Medicine, 200 First Street SW, Rochester, MN, 55905, USA

**Keywords:** Value-based purchasing (VBP) score, Clinical process of care, Patient satisfaction measure or Hospital Consumer Assessment of Healthcare Providers and Systems (HCAHPS) score, Health care quality, Safety-net hospitals, Quantile regression, Medicare

## Abstract

**Background:**

Medicare hospital Value-based purchasing (VBP) program that links Medicare payments to quality of care will become effective from 2013. It is unclear whether specific hospital characteristics are associated with a hospital’s VBP score, and consequently incentive payments.

The objective of the study was to assess the association of hospital characteristics with (i) the mean VBP score, and (ii) specific percentiles of the VBP score distribution. The secondary objective was to quantify the associations of hospital characteristics with the VBP score components: clinical process of care (CPC) score and patient satisfaction score.

**Methods:**

Observational analysis that used data from three sources: Medicare Hospital Compare Database, American Hospital Association 2010 Annual Survey and Medicare Impact File. The final study sample included 2,491 U.S. acute care hospitals eligible for the VBP program. The association of hospital characteristics with the mean VBP score and specific VBP score percentiles were assessed by ordinary least square (OLS) regression and quantile regression (QR), respectively.

**Results:**

VBP score had substantial variations, with mean score of 30 and 60 in the first and fourth quartiles of the VBP score distribution. For-profit status (vs. non-profit), smaller bed size (vs. 100–199 beds), East South Central region (vs. New England region) and the report of specific CPC measures (discharge instructions, timely provision of antibiotics and beta blockers, and serum glucose controls in cardiac surgery patients) were positively associated with mean VBP scores (p<0.01 in all). Total number of CPC measures reported, bed size of 400–499 (vs. 100–199 beds), a few geographic regions (Mid-Atlantic, West North Central, Mountain and Pacific) compared to the New England region were negatively associated with mean VBP score (p<0.01 in all). Disproportionate share index, proportion of Medicare and Medicaid days to total inpatient days had significant (p<0.01) but small effects. QR results indicate evidence of differential effects of some of the hospital characteristics across low-, medium- and high-quality providers.

**Conclusions:**

Although hospitals serving the poor and the elderly are more likely to score lower under the VBP program, the correlation appears small. Profit status, geographic regions, number and type of CPC measures reported explain the most variation among scores.

## Background

The Hospital Inpatient Value-Based Purchasing (VBP) program, enacted by the 2010 Patient Protection and Affordable Care Act (ACA) and effective in 2013, is a bold step towards “*transforming Medicare from a passive payer of claims to an active purchaser of quality health care for its beneficiaries*.” [1] Since the publication of Institute of Medicine’s reports on U.S. health care quality, various quality-improving initiatives, including Premier Hospital Quality Incentive Demonstration Project, have been undertaken by different stakeholders with only mixed results [2-5]. Modifiable gaps in the quality of care of U.S. hospitals still persist [6-8]. Well-documented large-scale regional variations in health care spending and service use among Medicare beneficiaries also signify lapses in the delivery of high value care [9,10]. Reducing these variations would potentially save 30-40% of Medicare budget [11,12], which constitutes 3.6% of the GDP and 15.1% of the annual U.S. federal budget [13].

The proposed VBP program will reward hospitals that provide better value, as assessed by the VBP score, which incorporates a mix of measures for process of care, outcomes, and patient-centeredness [1,14]. The VBP program is budget-neutral. The pool for incentive payments will be generated by holding back one percent of the base Medicare DRG payments to hospitals, which will then be used to reward the better-performing hospitals [1]. The worst-performing hospitals will not receive any VBP incentive payments, thus initially facing the prospect of losing one percent of Medicare payments. The holdback amount is slated to increase by 0.25 percentage points each subsequent year with a maximum at 2 percent from 2017 onwards. The estimated total 2013 VBP incentive payment is $850 million. Although the holdback does not seem huge for individual hospitals, the loss of even 1 percent of payments might have a significant negative impact on some hospital operations [13,15]. Moreover, future payment reductions, required by federal budget sequesters that further reduce the annual base DRG payment, will only intensify the financial pressure on hospitals with already small margins. It is also expected that commercial payers and Medicaid will follow Medicare’s lead, and begin linking payments to quality of care either measured through the VBP or another scoring system.

It is unclear as to whether hospital characteristics or the number and types of measures that hospitals report under the VBP program are associated with the estimated VBP score, and eventually influence the amount of incentive payments. Arguments have been raised that the VBP scoring scheme may unduly penalize hospitals that serve a higher proportion of minority and elderly patients [13,16]. Furthermore, it is important to understand whether specific hospital characteristics have differential impacts across low-, medium- and high-quality hospitals as reflected in the VBP score.

The primary objectives of the paper were to assess (i) the association between hospital characteristics and the mean VBP score, and (ii) the association between hospital characteristics and different percentiles of the VBP score, which will shed light on whether hospital characteristics have differential effects on low-, medium- and high-quality hospitals. The secondary objective was to assess the effects of hospital characteristics on the clinical process of care score and patient satisfaction score in order to understand whether the effects on the individual components translate to the total VBP score.

## Methods

### Data

The unit of analysis for our study is a U.S. hospital that is eligible for incentive payment under the VBP program. All Medicare Subsection (d) hospitals are eligible to participate in the VBP program, which includes all acute care hospitals in 50 states and District of Columbia other than rehabilitation hospitals and units; long-term care hospitals (LTCHs); psychiatric hospitals and units; children's hospitals; and cancer hospitals [1]. Furthermore, each of the hospitals must have at least 4 clinical process of care measures and at least 100 Hospital Consumer Assessment of Healthcare Providers and Systems (HCAHPS) surveys to be eligible for the VBP program. Critical Access hospitals are not included. [see Additional file 1: Appendix for further details]. Our data came from three primary sources: (i) Medicare Hospital Compare (HC) Access Database provided hospital specific information for all of the clinical process of care and patient experience measures; (ii) the 2010 American Hospital Association (AHA) Annual Survey database provided data characteristics including nurse staffing level, teaching status, and profit status; (iii) the 2009 Medicare Impact File provided information on the share of low-income patients served by a hospital and patient mix. [See Additional file 1: Appendix for further details] The authors have permission to use the AHA Survey dataset (which Mayo Clinic has purchased), while the other two datasets are publicly available.

### The performance threshold, the benchmark, and the VBP score calculation

A summary of the Medicare VBP program [1] is provided in Additional file 1: Appendix 1 in Supplementary Materials. In short, the VBP score for 2013 is based on a weighted average of either performance or improvement for 12 clinical process of care (CPC) measures and 8 patient satisfaction measures from HCAHPS survey. The HC database was used to extract the individual scores used for VBP score calculation for each hospital, the primary dependent variable in the analysis (Additional file 1: Appendix). The CPC and HCAHPS scores were dependent variables in the secondary analysis. We used performance threshold and benchmark for each measure as reported in the Federal Register [1]. Because HC database provides data only on a yearly basis, the baseline and the performance periods in our study comprised of full-year data as opposed to Medicare’s 3-quarter baseline and performance periods. More specifically, the baseline and performance periods for VBP score calculation in our study were from 4/1/2008 through 3/31/2009, and 4/1/2010 through 3/31/2011, respectively.

Table 1 shows the distribution of the estimated VBP score and its two components (CPC and HCAHPS Scores) overall and by 4 quartiles. The overall mean VBP score was 47, and there was wide variation in each of the scores as reflected by the range.

**Table 1:** Distribution of VBP, CPC and HCAHPS scores (Overall and by quartiles)

	**Mean**	**Median**	**Minimum**	**Maximum**	**SD**
**Overall VPB Score**	**47.1**	**46.0**	**2.0**	**99.0**	**14.4**
Quartile 1	29.9	31.0	2.0	37.0	6.4
Quartile 2	42.0	42.0	38.0	46.0	2.6
Quartile 3	51.0	51.0	47.0	56.0	2.8
Quartile 4	66.3	65.0	57.0	99.0	7.8
**Overall CPC Score**	50.7	50.0	0.0	100.0	18.2
Quartile 1	28.4	30.0	0.0	38.0	8.3
Quartile 2	44.6	45.0	39.0	50.0	3.5
Quartile 3	56.5	56.0	51.0	63.0	3.6
Quartile 4	74.9	73.0	64.0	100.0	8.7
**Overall HCAHPS Score**	38.6	35.0	5.0	100.0	17.9
Quartile 1	19.2	20.0	5.0	25.0	4.4
Quartile 2	30.6	31.0	26.0	35.0	2.9
Quartile 3	42.3	42.0	36.0	49.0	4.0
Quartile 4	63.8	60.0	50.0	100.0	12.5

### Hospital characteristics

Hospital characteristics assessed in the study are shown in Table 2, including Medicare-defined disproportionate share index (proportion of low income patients served by the hospital), teaching status, percents of Medicare and Medicaid days to total inpatient days, profit status, geographic region, total number and types of CPC measures reported. Detailed definitions of these variables are provided in Additional file 1: Appendix. Mean and standard deviation (SD) for continuous covariates, and the frequency and the percent for the categorical covariates are provided for the overall sample and for the four quartiles of the estimated VBP score.

**Table 2:** Descriptive characteristics of the hospitals (Overall and by four quartiles of the VBP score distribution)

**VARIABLES**	**OVERALL**		**QUARTILE 1**		**QUARTILE 2**		**QUARTILE 3**		**QUARTILE 4**		**P VALUE**
	**Mean**	**SD**	**Mean**	**SD**	**Mean**	**SD**	**Mean**	**SD**	**Mean**	**SD**	
CASE MIX INDEX	1.4	0.3	1.4	0.2	1.4	0.2	1.4	0.3	1.4	0.3	0.05
DISPROPORTIONATE SHARE PERCENT	26.7	16.3	29.5	19.1	28.0	15.7	25.0	14.2	24.1	14.9	0.00
PERCENT OF MEDICARE DAYS TO TOTAL INPATIENT DAYS	48.8	13.9	48.8	14.1	47.9	13.5	49.4	12.6	49.2	15.1	0.23
PERCENT OF MEDICAID DAYS TO TOTAL INPATIENT DAYS	19.4	13.3	22.2	15.5	20.5	13.0	18.4	12.3	16.3	11.3	0.00
PERCENT OF NURSE STAFFING LEVEL	79.6	42.0	74.3	34.3	75.9	26.5	78.8	31.8	89.6	63.5	0.00
TEACHING PERCENT	6.8	20.4	8.0	22.0	9.2	23.6	6.3	19.1	3.8	15.3	0.00
TOTAL NUMBER OF MEASURES REPORTED	9.7	1.3	9.8	1.3	9.9	1.1	9.8	1.2	9.3	1.6	0.00
	**n**	**%**	**n**	**%**	**n**	**%**	**n**	**%**	**n**	**%**	
PROFIT STATUS											0.000
FOR PROFIT	412	16.5	49	7.6	61	10.1	88	14.1	214	34.7	
NON-PROFIT	1707	68.5	466	72.3	441	72.8	456	73.2	344	55.8	
GOVERNMENT-OWNED(NON-FEDERAL)	372	14.9	130	20.2	104	17.2	79	12.7	59	9.6	
CATEGORIES OF NUMBER OF HOSPITAL BEDS											0.000
BEDS 6-49	244	9.8	44	6.8	46	7.6	52	8.4	102	16.5	
BEDS 50-99	477	19.2	113	17.5	102	16.8	123	19.7	139	22.5	
BEDS 100-199	783	31.4	229	35.5	171	28.2	191	30.7	192	31.1	
BEDS 200-299	429	17.2	114	17.7	116	19.1	107	17.2	92	14.9	
BEDS 300-399	253	10.2	57	8.8	77	12.7	65	10.4	54	8.8	
BEDS 400-499	130	5.2	43	6.7	42	6.9	32	5.1	13	2.1	
BEDS 500 OR MORE	175	7.0	45	7.0	52	8.6	53	8.5	25	4.1	
GEOGRAPHIC REGIONS											0.000
NEW ENGLAND	119	4.8	25	3.9	27	4.5	40	6.4	27	4.4	
MID ATLANTIC	301	12.1	94	14.6	91	15.0	77	12.4	39	6.3	
SOUTH ATLANTIC	453	18.2	111	17.2	96	15.8	123	19.7	123	19.9	
EAST NORTH CENTRAL	408	16.4	103	16.0	108	17.8	106	17.0	91	14.8	
EAST SOUTH CENTRAL	212	8.5	39	6.1	35	5.8	54	8.7	84	13.6	
WEST NORTH CENTRAL	213	8.6	55	8.5	61	10.1	54	8.7	43	7.0	
WEST SOUTH CENTRAL	370	14.9	79	12.3	82	13.5	77	12.4	132	21.4	
MOUNTAIN	148	5.9	48	7.4	38	6.3	31	5.0	31	5.0	
PACIFIC	267	10.7	91	14.1	68	11.2	61	9.8	47	7.6	
ACCREDITATION BY JCAHO	2146	86.2	536	83.1	529	87.3	545	87.5	536	86.9	0.080
OBSTETRIC CARE HOSPITAL	2072	83.2	565	87.6	531	87.6	524	84.1	452	73.3	0.000
WOUND MANAGEMENT SERVICES HOSPITAL	1914	76.8	521	80.8	495	81.7	490	78.7	408	66.1	0.000
MRI HOSPITAL	2221	89.2	564	87.4	551	90.9	573	92.0	533	86.4	0.000
GERIATRIC SERVICES HOSPITAL	1184	47.5	314	48.7	309	51.0	287	46.1	274	44.4	0.100
CLINICAL PROCESS OF CARE MEASURES											
FIBRINOLYTIC THERAPY WITHIN 30 MINUTES ON ARRIVAL	16	0.6	5	0.8	4	0.7	3	0.5	4	0.7	0.93
PRIMARY PCI WITHIN 90 MINUTES OF ARRIVAL	1219	48.9	320	49.6	336	55.5	317	50.9	246	39.9	0.00
PATIENTS GIVEN INSTRUCTIONS AT DISCHARGE	2420	97.2	632	98.0	603	99.5	616	98.9	569	92.2	0.00
BLOOD CULTURE PERFORMED IN EMERGENCY DEPARTMENT PRIOR TO INITIAL ANTIBIOTIC	2409	96.7	631	97.8	602	99.3	612	98.2	564	91.4	0.00
INITIAL ANTIBIOTIC SELECTION FOR ICU/NON-ICU PATIENTS	2420	97.2	634	98.3	606	100.0	611	98.1	569	92.2	0.00
PROPHYLACTIC ANTIBIOTIC GIVEN WITHIN 1 HOUR OF INCISION	2452	98.4	628	97.4	601	99.2	617	99.0	606	98.2	0.04
PROPHYLACTIC ANTIBIOTICS FOR SURGICAL PATIENTS	2451	98.4	628	97.4	602	99.3	616	98.9	605	98.1	0.03
ANTIBIOTICS DISCONTINUED WITHIN 24 HOURS AFTER SURGERY	2449	98.3	628	97.4	600	99.0	617	99.0	604	97.9	0.05
CARDIAC PATIENTS WITH CONTROLLED 6 AM POSTOPERATIVE SERUM GLUCOSE	1001	40.2	250	38.8	276	45.5	267	42.9	208	33.7	0.00
RECOMMENDED VTE PROPHYLAXIS ORDERED	2448	98.3	636	98.6	597	98.5	618	99.2	597	96.8	0.01
APPROPRIATE VTE PROPHYLAXIS WITHIN 24 HOURS OF SURGERY	2447	98.2	636	98.6	597	98.5	617	99.0	597	96.8	0.01
BETA BLOCKER PRIOR TO ADMISSION AND PERIOPERATIVELY	2379	95.5	605	93.8	587	96.86	603	96.79	584	94.65	0.02

Notes:

1. P-values for continuous covariates are based on ANOVA analysis and categorical variables are based on Chi-squared tests.

### Multivariable analyses

The association between the mean VBP score and hospital characteristics was assessed through ordinary least squared (OLS) regression. In selecting variables into the final model, besides including key variables that are expected to be associated with the estimated VBP score (e.g., DHS index, percent of Medicare and Medicaid days to total inpatient days, profit status, bed size, geographic regions), a stepwise forward selection with p-value less than 0.2 was adopted to decide on including other hospital characteristics. The final list of variables included in the model is shown in Table 2. We used conditional quantile regression (QR) to assess the association between various percentiles of the VBP score distribution and hospital characteristics [17,18]. Since hospitals in the upper tail (e.g., 90^th^ percentile) of the VBP score distribution are likely to receive the greatest incentive payments, while those in the lower tail (e.g., 10^th^ percentile or below) are likely to lose money under the VBP program, the QR approach offers insights on the potential determinants of the VBP scores for low-, medium and high-quality hospitals. Additionally, the QR approach helps assess whether a specific hospital characteristic has differential effects on hospitals across different parts of the VBP score distribution. (See Additional file 1: Appendix for further details).

### Study results

The final sample included 2,491 hospitals with complete observations for all the study variables. Table 2 provides descriptive statistics for hospital characteristics for the overall sample and for the four quartiles of the VBP score distribution.

Reported averages for disproportionate share index, percent of Medicaid inpatient days, and teaching status declined progressively from the first quartile (lower quality) to the fourth quartile (higher quality) of the VBP score distribution (Table 2). The opposite was true of the average nurse staffing level – the average nurse staffing in hospitals in the fourth quartile was 89 registered nurse full time employee (FTE) per 100 bed-days as compared to 74 in hospitals in the first quartile.

The average number of CPC measures reported was 9.7, with lesser mean number of CPC measures reported for 4^th^ quartile than the 1^st^ quartile (9.32 vs. 9.76). The distribution of hospitals by profit status across the four quartiles revealed an interesting pattern. The percent of for-profit hospitals increases as one moves from the 1^st^ to the 4^th^ quartile of the VBP score distribution; the opposite was true of government hospitals. The distribution of bed categories and geographic regions also differed significantly across the four VBP score quartiles.

### Multivariable results

**Figure 1:**
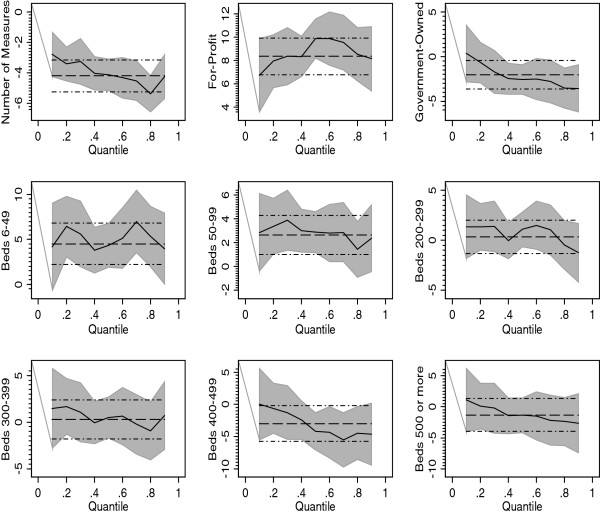
**Covariate Effects Under the Ordinary Least Squares and Quantile Regression Models.** The dashed straight line represents the OLS estimate while its 95% confidence interval is represented by dashed & dotted lines. The dark line represents quantile estimates at the 10 percentiles of the VBP score distributions (0.1, 0.2,.., 0.9), and their corresponding 95% confidence intervals are represented by the shaded area.

Table 3 presents the multivariable results associated with the OLS regression, and the QR estimated at five percentiles of the VBP score, namely, 10^th^, 25^th^, 50^th^, 75^th^ and 90^th^. Figure 1 exhibits the effects of some selected covariates along with their 95% confidence intervals, captured in the OLS and QR frameworks. For the purpose of these graphs, QR was estimated at 10 percentiles (0.1, 0.2, …, 0.9) of the VBP distribution.

**Table 3:** Regression estimates (Ordinary least squares and quantile regression estimates of estimated VBP score on hospital characteristics)

**VARIABLES**	**OLS**	**QUANTILE REGRESSION**				
		**Q10**	**Q25**	**Q50 (MEDIAN)**	**Q75**	**Q90**
DISPROPORTIONATE SHARE INDEX	−0.1***	−0.1**	−0.1***	−0.1***	−0.1**	−0.1**
	(−0.1, -0.0)	(−0.2, -0.0)	(−0.2, -0.1)	(−0.1, -0.0)	(−0.2, -0.0)	(−0.2, -0.0)
MEDICARE DAYS AS PERCENT OF INPATIENT DAYS	−0.1***	−0.1***	−0.2***	−0.1***	−0.1***	−0.2***
	(−0.2, -0.1)	(−0.2, -0.0)	(−0.2, -0.1)	(−0.2, -0.1)	(−0.2, -0.0)	(−0.3, -0.0)
MEDICAID DAYS AS PERCENT OF TOTAL INPATIENT DAYS	−0.1***	−0.1	−0.1***	−0.1**	−0.1***	−0.2***
	(−0.1, -0.0)	(−0.2, 0.0)	(−0.2, -0.0)	(−0.2, -0.0)	(−0.2, -0.0)	(−0.3, -0.1)
TOTAL NUMBER OF CPC MEASURES REPORTED	−4.1***	−2.4***	−3.6***	−3.6***	−4.7***	−4.3***
	(−5.1, -3.0)	(−3.7, -1.1)	(−4.6, -2.6)	(−5.0, -2.3)	(−6.0, -3.4)	(−5.9, -2.6)
PROFIT STATUS (REF: NON-PROFIT)						
FOR-PROFIT	7.9***	6.0***	8.3***	9.3***	8.3***	6.8***
	(6.3, 9.5)	(3.2, 8.9)	(6.3, 10.3)	(7.1, 11.6)	(5.9, 10.6)	(3.7, 9.9)
GOVERNMENT-OWNED (NON-FEDERAL)	−1.9**	0.1	−1.0	−2.5**	−3.0**	−3.8***
	(−3.5, -0.3)	(−2.8, 2.9)	(−2.9, 0.9)	(−4.7, -0.3)	(−5.4, -0.6)	(−6.6, -0.9)
BED CATEGORIES (REF: BEDS 100–199)						
BEDS 6-49	4.5***	4.2*	6.4***	4.9***	5.4***	4.7**
	(2.1, 6.8)	(−0.1, 8.5)	(3.5, 9.4)	(1.6, 8.2)	(1.9, 8.9)	(0.3, 9.2)
BEDS 50-99	2.7***	2.9**	4.2***	3.0**	1.4	2.0
	(1.0, 4.4)	(0.0, 5.9)	(2.1, 6.2)	(0.7, 5.3)	(−1.0, 3.9)	(−1.1, 5.1)
BEDS 200-299	0.2	1.3	0.9	0.6	0.2	−1.5
	(−1.4, 1.9)	(−1.5, 4.1)	(−1.1, 2.9)	(−1.7, 3.0)	(−2.3, 2.7)	(−4.6, 1.7)
BEDS 300-399	0.3	2.3	1.5	0.2	−0.8	−0.1
	(−1.8, 2.4)	(−1.4, 6.0)	(−1.0, 4.1)	(−2.8, 3.1)	(−4.0, 2.4)	(−4.1, 4.0)
BEDS 400-499	−3.0**	−0.9	−0.7	−3.5*	−4.8**	−4.9*
	(−5.8, -0.2)	(−5.9, 4.1)	(−4.2, 2.7)	(−7.4, 0.4)	(−9.0, -0.5)	(−10.1, 0.4)
BEDS 500 OR MORE	−1.4	−0.1	0.1	−1.9	−1.6	−2.6
	(−4.1, 1.3)	(−4.7, 4.5)	(−3.1, 3.4)	(−5.7, 1.9)	(−5.6, 2.4)	(−7.8, 2.5)
GEOGRAPHIC REGION (REF: NEW ENGLAND)						
MID ATLANTIC	−3.2**	−3.4	−3.2*	−4.2**	−3.0	−3.6
	(−6.0, -0.4)	(−8.3, 1.5)	(−6.6, 0.3)	(−8.0, -0.3)	(−7.2, 1.1)	(−8.7, 1.5)
SOUTH ATLANTIC	0.8	−2.0	−1.3	−0.4	3.8*	4.3*
	(−1.9, 3.5)	(−6.8, 2.8)	(−4.6, 2.1)	(−4.1, 3.4)	(−0.3, 7.8)	(−0.6, 9.3)
EAST NORTH CENTRAL	−1.1	−3.5	−1.6	−2.2	0.8	1.2
	(−3.8, 1.6)	(−8.2, 1.2)	(−4.9, 1.7)	(−5.9, 1.6)	(−3.2, 4.9)	(−3.8, 6.2)
EAST SOUTH CENTRAL	4.1***	0.4	2.8	3.7*	7.9***	6.5**
	(1.0, 7.2)	(−4.8, 5.6)	(−0.9, 6.5)	(−0.6, 8.0)	(3.3, 12.6)	(0.7, 12.2)
WEST NORTH CENTRAL	−3.1**	−4.3	−4.2**	−3.2	−2.5	−1.4
	(−6.1, -0.1)	(−9.6, 0.9)	(−7.8, -0.5)	(−7.4, 1.0)	(−7.0, 2.0)	(−7.0, 4.2)
WEST SOUTH CENTRAL	−0.0	−2.1	−2.1	−1.2	2.6	3.5
	(−2.9, 2.9)	(−7.2, 3.0)	(−5.7, 1.4)	(−5.3, 2.8)	(−1.7, 6.9)	(−1.8, 8.7)
MOUNTAIN	−5.5***	−7.8***	−6.6***	−6.9***	−3.7	−0.4
	(−8.8, -2.2)	(−13.7, -2.0)	(−10.6, -2.6)	(−11.5, -2.3)	(−8.6, 1.2)	(−6.4, 5.5)
PACIFIC	−3.9***	−6.9***	−4.9***	−5.3**	−2.7	−1.3
	(−6.9, -0.9)	(−12.2, -1.7)	(−8.5, -1.2)	(−9.5, -1.2)	(−7.1, 1.7)	(−6.7, 4.1)
WHETHER JCAHO ACCREDITED	−2.5***	−5.0***	−2.8***	−2.7**	−0.9	−1.1
	(−4.1, -1.0)	(−7.8, -2.2)	(−4.7, -0.9)	(−4.9, -0.5)	(−3.3, 1.4)	(−4.0, 1.8)
OBSTETRIC CARE HOSPITAL	−2.3***	−0.4	−1.5	−3.4***	−2.4**	−2.7*
	(−3.8, -0.8)	(−3.2, 2.3)	(−3.5, 0.4)	(−5.6, -1.3)	(−4.7, -0.1)	(−5.7, 0.3)
GERIATRIC SERVICES HOSPITAL	1.1**	0.6	0.3	0.9	1.2	1.2
	(0.0, 2.2)	(−1.4, 2.5)	(−1.1, 1.7)	(−0.7, 2.4)	(−0.4, 2.9)	(−0.9, 3.2)
MRI HOSPITAL	1.5*	0.7	2.3**	1.9	1.0	2.5
	(−0.2, 3.2)	(−2.4, 3.7)	(0.2, 4.5)	(−0.5, 4.3)	(−1.5, 3.5)	(−0.7, 5.7)
WOUND MANAGEMENT SERVICES HOSPITAL	−1.6**	−1.4	−1.8**	−1.5	−1.7	−1.7
	(−3.0, -0.3)	(−3.8, 0.9)	(−3.5, -0.1)	(−3.4, 0.4)	(−3.6, 0.3)	(−4.2, 0.8)
PRIMARY PCI WITHIN 90 MINUTES OF ARRIVAL	2.8***	2.3	3.8***	2.9**	2.4	2.2
	(0.8, 4.9)	(−1.0, 5.5)	(1.4, 6.2)	(0.1, 5.7)	(−0.5, 5.4)	(−1.6, 5.9)
PATIENTS GIVEN INSTRUCTIONS AT DISCHARGE	9.1***	5.7*	13.5***	8.9***	9.7***	6.9
	(4.7, 13.5)	(−0.5, 12.0)	(8.7, 18.3)	(3.2, 14.6)	(3.5, 15.9)	(−1.8, 15.5)
PROPHYLACTIC ANTIBIOTIC GIVEN WITHIN 1 HOUR OF INCISION	11.1***	13.4***	12.7***	12.8***	14.6***	−2.2
	(6.1, 16.0)	(5.7, 21.2)	(7.5, 17.9)	(6.3, 19.3)	(7.8, 21.5)	(−9.9, 5.5)
CARDIAC PATIENTS WITH CONTROLLED 6 AM POSTOPERATIVE SERUM GLUCOSE	5.0***	3.9**	4.2***	4.4***	5.0***	4.7**
	(2.8, 7.2)	(0.3, 7.5)	(1.5, 6.9)	(1.4, 7.5)	(1.9, 8.2)	(0.8, 8.7)
BETA BLOCKER PRIOR TO ADMISSION AND PERIOPERATIVELY	7.7***	6.6**	9.1***	6.8***	7.8***	7.1**
	(4.4, 11.0)	(1.1, 12.1)	(5.2, 12.9)	(2.4, 11.2)	(3.1, 12.6)	(1.7, 12.6)
Constant	69.2***	41.5***	48.2***	66.0***	76.4***	107.9***
	(58.5, 79.8)	(23.0, 59.9)	(34.7, 61.7)	(51.4, 80.6)	(60.7, 92.1)	(87.4, 128.3)
Observations	2,491	2,491	2,491	2,491	2,491	2,491
R-squared	0.2					

Notes:

1. 95% Confidence Intervals in parentheses.

2. *** p<0.01, ** p<0.05, * p<0.1.

Case-mix index, nurse staffing ratio and teaching level had non-significant effects both in OLS and all of QR estimates (not shown in Table 3 to avoid cluttering). Disproportionate share index, percents of Medicare and Medicaid inpatient days were inversely associated with the VBP score. As seen from the corresponding quantile estimates, these covariates had uniform effects across all the 5 percentiles considered.

The total number of CPC measures reported was significantly inversely associated with the mean VBP score, and this effect was increasingly more pronounced going from lower to upper quantiles of the VBP distribution. Compared with non-profit hospitals, for-profit hospitals were likely to have significantly higher mean VBP score. The significant positive association between for-profit status and the VBP score was observed across the entire VBP score distribution. Government hospitals were likely to have lower mean VBP scores and the effects get more pronounced in the uppermost quantile (i.e., 90^th^ percentile).

Compared with hospitals with 100–199 beds, hospitals with fewer beds had significantly higher VBP score, while those hospitals with 400 or more beds tended to have smaller VBP scores. The analysis also found significant regional differences. Compared to the New England region, hospitals located in Mid Atlantic, West North Central, Mountain and Pacific regions had significantly lower mean VBP scores, while those located in East South Central had significantly higher mean VBP scores. QR estimates revealed heterogeneous effects of geographic location across the VBP score distribution. On average, accreditation by the Joint Commission, hospitals providing obstetric care or wound management services were negatively associated with the VBP score.

Hospitals that reported the following 4 specific CPC measures were likely to have significantly higher mean VBP scores (Table 3): primary PCI within 90 minutes of arrival, prophylactic antibiotic given with 1 hour of incision, cardiac patients with controlled 6 AM postoperative serum glucose, and beta blocker prior to admission and perioperatively. Note however, that these effects got progressively diluted towards the upper quantiles of the VBP score distribution.

The heterogeneous effects of some selected hospital characteristics across different parts of the VBP score distribution are shown in Figure 1, which overlays the quantile estimates with the OLS estimates along with their 95% confidence intervals. As Figure 1 shows, the quantile effects of the number of CPC measures reported was less than the mean effect in the lower tail of the VBP score distribution; however, the effect was substantially higher than the mean effects in the upper tail. The differential effects of profit status and bed size are shown in the other panels of Figure 1. (See Additional file 1: Appendix for geographic regions and percent of Medicare inpatient days)

### Association of covariates with the components of VBP score

This sub-analysis addressed the secondary study objective, and was based on the QR framework. The results are shown only for the 90^th^ percentile of the VBP score (Table 4). The coefficient for the QR effect of case-mix index on the 90^th^ percentile of the HCAHPS score was 9, while that for CPC score was −8, both statistically significant. These opposing effects resulted in a non-significant net effect of 2.1 on the 90^th^ percentile of the VBP score. The number of CPC measures reported was negatively associated with both CPC and HCAHPS scores, which translated to a net negative effect on the VBP score.

**Table 4:** **Quantile regression estimates of CPC, HCAHPS and VBP scores at 90**^**th **^**percentile**

	**(1)**	**(2)**	**(3)**
**VARIABLES**	**CPC**	**HCAHPS**	**VBP**
CASE MIX INDEX	−7.7***	8.8**	−2.1
	(−13.4, -2.0)	(2.0, 15.6)	(−8.0, 3.9)
DISPROPORTIONATE SHARE PERCENT	−0.0	−0.1**	−0.1**
	(−0.1, 0.0)	(−0.2, -0.0)	(−0.2, -0.0)
PERCENT OF MEDICARE TO TOTAL INPATIENT DAYS	−0.1***	0.0	−0.2***
	(−0.2, -0.0)	(−0.1, 0.1)	(−0.3, -0.0)
PERCENT OF MEDICAID TO TOTAL INPATIENT DAYS	−0.2***	0.0	−0.2***
	(−0.3, -0.1)	(−0.1, 0.1)	(−0.3, -0.1)
PERCENT OF NURSE STAFFING LEVEL	−0.0**	0.1***	−0.0
	(−0.1, -0.0)	(0.0, 0.1)	(−0.0, 0.0)
TEACHING PERCENT	0.0	−0.0	−0.0
	(−0.0, 0.1)	(−0.1, 0.1)	(−0.1, 0.0)
TOTAL NUMBER OF MEASURES REPORTED	−4.5***	−3.6***	−4.3***
	(−6.1, -3.0)	(−5.0, -2.3)	(−5.9, -2.6)
PROFIT STATUS (REF: NON-PROFIT)			
FOR-PROFIT	10.1***	−4.2***	6.8***
	(7.2, 13.0)	(−7.3, -1.1)	(3.7, 9.9)
GOVERNMENT-OWNED (NON-FEDERAL)	−6.3***	1.0	−3.8***
	(−9.1, -3.6)	(−2.2, 4.2)	(−6.6, -0.9)
BED CATEGORIES (REF: BEDS 100–199)			
BEDS 6-49	0.6	10.7***	4.7**
	(−3.5, 4.7)	(5.6, 15.8)	(0.3, 9.2)
BEDS 50-99	−2.3	8.0***	2.0
	(−5.2, 0.6)	(4.6, 11.3)	(−1.1, 5.1)
BEDS 200-299	−1.2	−6.2***	−1.5
	(−4.1, 1.7)	(−9.5, -2.9)	(−4.6, 1.7)
BEDS 300-399	1.6	−4.8**	−0.1
	(−2.1, 5.4)	(−8.8, -0.8)	(−4.1, 4.0)
BEDS 400-499	−6.3**	−8.1***	−4.9*
	(−11.2, -1.4)	(−13.6, -2.6)	(−10.1, 0.4)
BEDS 500 OR MORE	−1.7	−12.2***	−2.6
	(−6.4, 2.9)	(−17.5, -7.0)	(−7.8, 2.5)
GEOGRAPHIC REGION (REF: NEW ENGLAND)			
MID ATLANTIC	0.1	−11.4***	−3.6
	(−4.8, 5.0)	(−16.8, -6.0)	(−8.7, 1.5)
SOUTH ATLANTIC	5.8**	4.9*	4.3*
	(1.2, 10.5)	(−0.3, 10.1)	(−0.6, 9.3)
EAST NORTH CENTRAL	1.7	−1.3	1.2
	(−3.0, 6.4)	(−6.6, 4.1)	(−3.8, 6.2)
EAST SOUTH CENTRAL	6.6**	11.5***	6.5**
	(1.3, 11.9)	(5.4, 17.5)	(0.7, 12.2)
WEST NORTH CENTRAL	−2.5	−1.0	−1.4
	(−7.7, 2.7)	(−7.1, 5.1)	(−7.0, 4.2)
WEST SOUTH CENTRAL	4.1	2.6	3.5
	(−0.8, 9.1)	(−3.1, 8.3)	(−1.8, 8.7)
MOUNTAIN	1.3	−6.2*	−0.4
	(−4.2, 6.9)	(−12.8, 0.5)	(−6.4, 5.5)
MID ATLANTIC	3.4	−10.0***	−1.3
	(−1.5, 8.4)	(−16.0, -4.1)	(−6.7, 4.1)
ACCREDITATION BY JCAHO	−1.7	0.6	−1.1
	(−4.4, 1.1)	(−2.5, 3.7)	(−4.0, 1.8)
OBSTETRIC CARE HOSPITAL	−3.0**	−2.3	−2.7*
	(−5.7, -0.3)	(−5.6, 1.0)	(−5.7, 0.3)
WOUND MANAGEMENT SERVICES HOSPITAL	−2.4**	−3.1**	−1.7
	(−4.8, -0.0)	(−5.8, -0.4)	(−4.2, 0.8)
MRI HOSPITAL	2.1	0.0	2.5
	(−0.9, 5.2)	(−3.3, 3.4)	(−0.7, 5.7)
GERIATRIC SERVICES HOSPITAL	3.1***	1.3	1.2
	(1.2, 5.1)	(−1.0, 3.6)	(−0.9, 3.2)
PRIMARY PCI WITHIN 90 MINUTES OF ARRIVAL	2.2	0.2	2.2
	(−1.2, 5.6)	(−3.6, 4.0)	(−1.6, 5.9)
PATIENTS GIVEN INSTRUCTIONS AT DISCHARGE	8.3**	−5.6	6.9
	(1.1, 15.5)	(−14.9, 3.7)	(−1.8, 15.5)
PROPHYLACTIC ANTIBIOTIC GIVEN WITHIN 1 HOUR OF INCISION	−6.1	−2.3	−2.2
	(−13.8, 1.7)	(−10.2, 5.6)	(−9.9, 5.5)
CARDIAC PATIENTS WITH CONTROLLED 6 AM POSTOPERATIVE SERUM GLUCOSE	4.9***	6.1***	4.7**
	(1.2, 8.5)	(2.2, 10.1)	(0.8, 8.7)
BETA BLOCKER PRIOR TO ADMISSION AND PERIOPERATIVELY	10.9***	5.1*	7.1**
	(5.3, 16.5)	(−0.6, 10.7)	(1.7, 12.6)
Constant	127.4***	81.3***	107.9***
	(108.9, 145.9)	(59.5, 103.1)	(87.4, 128.3)

Notes:

1. 95% Confidence Intervals in parentheses.

2. *** p<0.01, ** p<0.05, * p<0.1.

Profit status had opposite effects on HCAHPS and CPC scores at the 90^th^ percentile. Compared with non-profit hospitals, for-profit hospitals had negative effects (4 units) on the HCAHPS score but positive (10 units) on the CPC score with a net effect of 7 units on the total VBP score. Government hospitals had no significant effect on HCAHPS score, although it had significantly negative effect on the CPC score that was mediated to the total VBP score.

## Discussion

The association between hospital characteristics and quality of care was assessed previously using 10 of the reported measures by Hospital Quality Alliance [19]. Jha and colleagues recently found that the “worst” hospitals cared for disproportionately higher numbers of Medicaid and black elderly patients than the “best” hospitals [20]. Lehrman et al. identified key hospital characteristics that determine whether the hospital will perform in the top quartile on both CPC and patient satisfaction measures [21]. However, results from these studies may not be directly applicable to ongoing policy discussions as they were based on quality measures constructed by the authors. While reasonable, policy conclusions based on these measures could, at best, be only suggestive but not directly applied to drive future policy. Our study, based on the closest possible approximation of the VBP score, adds value to the ongoing debate by quantifying how a wide range of hospital characteristics may be potentially associated with the VBP score. The study found that profit status (18%), geographic region (12%), and the number of reported CPC measures (9%) explained the most variation in the estimated VBP score (see Additional file 1: Appendix for the percent of variance explained for other hospital characteristics). The study also provided evidence of heterogeneous effects of some hospital characteristics on low-, medium- and high-quality hospitals.

Disproportionate share index, a rough proxy for “safety-net” hospitals serving larger percentages of low-income people, was negatively associated with VBP scores; however, its marginal effect on the VBP score was only 1/10^th^ of a unit score. Similarly, the proportion of Medicare and Medicaid inpatient days were negatively associated with VBP score but the estimated marginal effects were rather small. Although hospitals treating higher percentages of Medicare patients are presumed to be worried about the VBP program’s impact on their reimbursement, our data suggest that any impact would be rather small. Furthermore, there is little variation of this effect across the VBP score distribution as evidenced in both Tables 2 and 3. These findings would downplay the potential unintended consequences of VBP, which speculate that hospitals serving low-income patients, Medicare and Medicaid patients are at a disadvantage to compete with high-performing hospitals [22].

We found that many hospital characteristics were associated with the mean VBP score (Table 3), yet the measures relevant for VBP incentive payments are those that are associated with the higher quantiles (e.g., 90^th^ percentile) of the VBP score distribution. The number of CPC measures reported was negatively associated with the mean VBP score, and as the QR estimates suggest this effect increased monotonically from lower to higher quantiles of the VBP score. This potentially suggests that the 90^th^ percentile of the VBP score will be lower for large multispecialty hospitals that typically report higher number of CPC measures than smaller super-specialty hospitals that report fewer CPC measures. The finding that for-profit hospitals have significantly higher VBP scores than non-profit hospitals (and hence higher quality) contradicts a recent survey of literature that found non-profit hospitals to provide better-quality care than for-profit hospitals [23]. However, note that most studies that assessed the association between quality of care and profit-status used mortality as a proxy for quality, which is often the driving force in lower quality in for-profit hospitals [23,24]. Mortality is not included in the 2013 VBP score calculation [1,23], which might explain this contradictory finding. Contrary to the positive association of nurse staffing and teaching level with quality [19], our study did not find any such evidence.

In general, hospital bed size was found to be negatively associated with the VBP score. Our study confirmed well-documented regional variations in care quality [25]. However, at the 90^th^ percentile of VBP score, only South Atlantic and East South Central regions had significant positive effects on the VBP score than New England region. The mean VBP score was also significantly correlated with the type of CPC measures reported, including whether heart failure patients were given instruction at discharge and whether prophylactic antibiotic was given within 1 hour of incision for surgical patients.

The effects of hospital characteristics on each of the component domains of the VBP score, CPC and patient satisfaction scores, provides important insights on how the net effect on the VBP score might be related to the component effects (Table 4). For example, the net effect of 6–49 bed category on the total VBP score was primarily driven by its association with the patient experience score. Consistent with the earlier finding [26], although nurse staffing and for-profit status were found to have positive and negative association with patient satisfaction score, respectively, these associations were not strong enough to influence the resulting total VBP score.

VBP is an evolving measure, and Medicare is going to include other measures (outcomes measures including 30-day mortality rate for AMI, heart failure and pneumonia) in 2014, and safety and efficiency measures in the subsequent years. This evolving nature of the VBP program makes it difficult to predict how hospitals will respond.

There are several limitations to our analysis. Because Hospital Compare website reports only annual data, our analysis could differ from official VBP results calculated using 3-quarter baseline and performance periods. However, for most institutions, this difference is not expected to be significant. Our analysis also uses data from one year prior to VBP implementation. Although individual hospitals may have different performance, one would not expect the associations of VBP scores with major hospital characteristics to differ significantly in one year. Furthermore, since the analyses were based on historical data, our study does not take into account the possibility that the hospital might behave differently once the VBP incentives kick in, and hence the associations between hospital characteristics and the VBP score predicted in our study may be different. However, note that structural characteristics such as bed size or for-profit status are difficult to change in the short run, and thus we anticipate that the overall findings of our study will not change at least in the next few years after the proposed VBP implementation in 2013.

### Policy implications

Our findings allay the concerns that hospitals serving the poor and the elderly are more likely to score lower and hence, likely to be penalized under the VBP program. However, the loss of 1% VBP revenue could significantly influence the future viability of these hospitals that operate under very tight financial constraints. Given that for-profit status explains the most of the variation in the VBP score and potentially the incentive payments, we might witness a gradual change in ownership in the long run. This possibility becomes more real when commercial payers and Medicaid follow suit in adopting value-based purchasing or similar measures. The 2010 ACA has already called for extending value-based purchasing to physician payment [27]. In order to receive highest incentive payments, and remain financially viable, hospitals may have to make structural changes including ownership and types of services offered. As the report to the Congress suggested, it will be of utmost importance to monitor if hospitals deny care to specific groups of patients simply to maintain a high VBP score [14].

## Conclusion

Our analysis finds significant association between several hospital characteristics and the total VBP score. In particular, profit-status, geographic location, total number and types of CPC measures reported, and hospital size were found to be significantly associated with the VBP score.

## Abbreviations

VBP: Value-based purchasing; OLS: Ordinary Least Square; QR: Quantile Regression; CPC: Clinical Process Of Care; HCAHPS: Hospital Consumer Assessment of Healthcare Providers and Systems; ACA: 2010 Patient Protection and Affordable Care Act; GDP: Gross Domestic Product; DRG: Diagnosis-related Group; HC: Medicare Hospital Compare; SD: Standard Deviation.

## Competing interests

None of the authors have any conflict of interests to declare.

## Authors’ contributions

BJB, MGR, DLW and JMN contributed to concept, design and critical revision of the manuscript; BJB, DLR and MGJ contributed to data acquisition and constructing the analytic data file; BJB and JMN contributed to the analysis and interpretation of the data; BJB and JMN contributed to drafting of the manuscript. BJB has full access to all the data used in the study, and takes responsibility for the integrity of the data and the accuracy of the data analysis. The study was internally funded by Mayo Clinic. However, Mayo Clinic has not influenced the study design, findings and interpretations. All authors read and approved the final manuscript.

## Pre-publication history

The pre-publication history for this paper can be accessed here:

http://www.biomedcentral.com/1472-6963/12/464/prepub

## Supplementary Material

Additional file 1 Appendix 1: The 2013 VBP Program Summary and Appendix 2: Description of the Data Sources. Click here for file

## References

[B1] Center for Medicare and Medicaid Services Medicare program; hospital inpatient value-based purchasing program, 76 FR 26,490 Federal register, Vol 76, No. 882011 2011 Washington, D.C.: Government Printing Office (GPO) 21548401

[B2] Institute of Medicine To err is human: building a safer health system 1999 Washington, D.C.: National Academy Press

[B3] Institute of Medicine Crossing the quality chasm: a new health system for the twenty-first century 2001 Washington, D.C.: National Academy Press

[B4] Dentzer S Still crossing the quality chasm-or suspended over it? Health Aff (Millwood) 2011 30 4 554 555 [Editorial Introductory] 10.1377/hlthaff.2011.0287 21471471

[B5] Centers for Medicare & Medicaid Services The premier hospital quality incentive demonstration project 2011 Baltimore, Maryland: CMS cited 2011 December 14

[B6] Chassin MR Loeb JM The ongoing quality improvement journey: next stop, high reliability Health Aff (Millwood) 2011 30 4 559 568 [Historical Article] 10.1377/hlthaff.2011.0076 21471473

[B7] Fowler FJ Jr Levin CA Sepucha KR Informing and involving patients to improve the quality of medical decisions Health Aff (Millwood) 2011 30 4 699 706 10.1377/hlthaff.2011.0003 21471491

[B8] Maynard A The powers and pitfalls of payment for performance Heal Econ 2012 21 3 12 10.1002/hec.1810 22147622

[B9] Medicare Payment Advisory Commission (MEDPAC) Report to the congress: measuring regional variation in service use 2009 Washington, D.C.: MEDPAC

[B10] Medicare Payment Advisory Commission (MEDPAC) Report to the congress: regional variation in Medicare service use 2011 Washington, D.C.: MEDPAC

[B11] Fisher ES Medical care-is more always better? N Engl J Med 2003 349 17 1665 1667 [Comment Editorial] 10.1056/NEJMe038149 14573739

[B12] Wennberg JE Tracking medicine: a researchers's quest to understand health care 2010 New York: Oxford University Press

[B13] Kaiser Family Foundation (KFF) Medicare spending and financing: a premier 2011 Menlo Park, CA: KFF

[B14] Center for Medicare and Medicaid Services Report to congress: plan to implement a Medicare hospital value-based purchasing program 2007 Baltimore, Maryland: CMS

[B15] Werner RM Goldman LE Dudley RA Comparison of change in quality of care between safety-net and non-safety-net hospitals JAMA 2008 299 18 2180 2187 [Comparative Study Research Support, Non-U.S. Gov't Research Support, U.S. Gov't, Non-P.H.S. Research Support, U.S. Gov't, P.H.S.] 10.1001/jama.299.18.2180 18477785

[B16] Jha AK Orav EJ Zheng J Epstein AM The characteristics and performance of hospitals that care for elderly Hispanic Americans Health Aff (Millwood) 2008 27 2 528 537 [Research Support, Non-U.S. Gov't] 10.1377/hlthaff.27.2.528 18332511

[B17] Koenker R Quantile regression 2005 New York: Cambridge University Press

[B18] Koenker R Hallock KF Quantile regression J Econ Perspect 2001 15 4 143 156 10.1257/jep.15.4.143

[B19] Jha AK Li Z Orav EJ Epstein AM Care in U.S. Hospitals-the hospital quality alliance program N Engl J Med 2005 353 3 265 274 [Research Support, Non-U.S. Gov't] 10.1056/NEJMsa051249 16034012

[B20] Jha AK Orav EJ Epstein AM Low-quality, high-cost hospitals, mainly in south, care for sharply higher shares of elderly black, Hispanic, and Medicaid patients Health Aff (Millwood) 2011 30 10 1904 1911 [Research Support, Non-U.S. Gov't] 10.1377/hlthaff.2011.0027 21976334

[B21] Lehrman WG Elliott MN Goldstein E Beckett MK Klein DJ Giordano LA Characteristics of hospitals demonstrating superior performance in patient experience and clinical process measures of care Med Care Res Rev 2010 67 1 38 55 [Research Support, Non-U.S. Gov't] 10.1177/1077558709341323 19638640

[B22] Joynt KE Rosenthal MB Hospital value-based purchasing: will Medicare's New policy exacerbate disparities? Circ Cardiovasc Qual Outcomes 2012 5 2 148 149 10.1161/CIRCOUTCOMES.112.965178 22438462

[B23] Eggleston K Shen YC Lau J Schmid CH Chan J Hospital ownership and quality of care: what explains the different results in the literature? Health Econ 2008 17(12 1345 1362 [Meta-Analysis Research Support, Non-U.S. Gov't] 18186547 10.1002/hec.1333

[B24] McClellan M Staiger D Cutler D Comparing hospital quality at for-profit and not-for-profit hospitals The changing hospital industry: comparing for-profit and Not-for-profit institutions 2000 Chicago: University of Chicago Press 93 112

[B25] Dartmouth Institute of Health Policy & Clinical Practice The Dartmouth atlas of health care 2011 cited 2011 December 14]; Available from: http://www.dartmouthatlas.org. 36516284

[B26] Jha AK Orav EJ Zheng J Epstein AM Patients' Perception of hospital care in the United States N Engl J Med 2008 359(18 1921 1931 [Research Support, Non-U.S. Gov't] 18971493 10.1056/NEJMsa0804116

[B27] Ginsburg PB Rapidly evolving physician-payment policy-more than the SGR N Engl J Med 2011 364 2 172 176 [Research Support, Non-U.S. Gov't] 10.1056/NEJMhpr1004028 21142529

