# Supplementary material for: Association between value-based purchasing score and hospital characteristics
Source: BMC Health Serv Res. 2012 Dec 17;12:464. doi: 10.1186/1472-6963-12-464 (PMC3557150; doi:10.1186/1472-6963-12-464)
Supplement: Additional file 1 — Appendix 1: The 2013 VBP Program Summary and Appendix 2: Description of the Data Sources. [file 1472-6963-12-464-S1.doc]

**Appendix 1: The 2013 VBP Program Summary**

The first phase of the VBP program is set to apply to discharges of Medicare patients occurring on or after October 1, 2012. The first phase, however, will include only two domains: clinical process of care (CPC) and patient experience of care. The third domain, outcome measures, will be incorporated in the VBP formula from fiscal year 2014 onwards, which will initially include three 30-day mortality measures. The CPC domain incorporates twelve clinical process of care measures covering best practices in acute myocardial infection (AMI), heart failure (HF), pneumonia (PN), and surgical care improvement project (SCIP) (See Appendix 1 Exhibit 1 in Supplementary Materials). The patient experience of care domain incorporates eight dimensions, as captured by Hospital Consumer Assessment of Healthcare Providers and Systems Survey (HCAHPS) (See Appendix 1 Exhibit 2 in Supplementary Materials). The baseline assessment period for the 2013 VBP program for these 20 measures will be the 3-quarter period between July 1, 2009 through March 31, 2010, while the performance period will be the 3-quarter period starting from July 1, 2011 through March 31, 2012. For each measure, both achievement (compared to the national distributions) and improvement (change from the hospital’s own baseline performance) will be considered, with the higher of the two used in the calculation of the overall VBP score. For any hospital, achievement in a particular measure will be based on where its performance falls between the achievement threshold (national median) and the benchmark. For clinical process of care, the benchmark is defined as the mean of the top decile of the hospital scores on that measure, while for patient satisfaction measures, the benchmark is defined as the 95^th^ percentile of the performance score among the US hospitals. The improvement score for both process and patient experience measures is calculated relative to a hospital’s own performance on that measure during the baseline period. For the patient experience of care domain, in addition to the eight dimension scores, a hospital may be awarded up to 20 consistency points based on where the lowest of the eight HCAHPS dimension scores falls vis-à-vis the achievement threshold for that dimension. The overall domain score for clinical process of care measures is calculated as the percentage of total earned points to the total possible points from all the measures in this domain for which the hospital qualifies. The overall patient experience score is the sum of all 8 HCAHPS dimension scores plus the consistency score. The total VBP score for a hospital, which is used for determining incentive payments, is then determined by aggregating the two domain scores, with 70 percent weight assigned to the clinical process of care domain and 30 percent weight assigned to the patient experience of care domain. Note that in order for a measure to be included in a hospital VBP score, at least 10 cases must be reported on that measure. The VBP score will then be translated into an incentive amount based on a linear exchange function, the slope of which will be determined so that the incentive payments under the VBP program remain budget neutral. Hospitals reporting fewer than 4 clinical process of care measures or fewer than 100 HCAHPS surveys will be excluded from the 2013 VBP program and will not incur a reduction in their base operating DRG amounts.

Appendix Exhibit 1: VBP Component Measures

| **Clinical Process Measures** | |
| --- | --- |
| **Variable Names** | **Description** |
| AMI_7a | Fibrinolytic therapy within 30 minutes or arrival |
| AMI_8a | Primary PCI within 90 minutes of arrival |
| HF_1 | Patients given instructions at discharge |
| PN_3b | Blood culture performed in ED prior to initial antibiotic |
| PN_6 | Initial antibiotic selection for ICU/non-ICU patients |
| SCIP_INF_1 | Prophylactic antibiotic given within 1 hour of incision |
| SCIP_INF_2 | Prophylactic antibiotics for surgical patients |
| SCIP_INF_3 | Antibiotics discontinued within 24 hours after surgery |
| SCIP_INF_4 | Cardiac patients with controlled 6 am postop serum glucose |
| SCIP_VTE_1 | Recommended VTE Prophylaxis ordered |
| SCIP_VTE_2 | Appropriate VTE Prophylaxis within 24 hours of surgery |
| SCIP_CARD_2 | Beta blocker prior to admission and periop |
| **Patient Satisfaction (HCAHPS) Measures** | |
| H_COMP_1_A_P | How well nurses communicated with patient |
| H_COMP_2_A_P | How well doctors communicated with patient |
| H_COMP_3_A_P | Responsiveness of staff |
| H_COMP_4_A_P | How well was pain managed by staff |
| H_COMP_5_A_P | How well staff communicated medicine information |
| H_CLEAN_QUIET_HSP_A_P | Hospital cleanliness & quietness |
| H_COMP_6_Y_P | Information given at discharge |
| H_HSP_RATING_9_10 | Overall rating of hospital |

**Appendix 2: Description of the Data Sources**

***Medicare Hospital Compare (HC) Database***

The Hospital Compare database is a joint initiative of Centers for Medicare & Medicaid Services (CMS) and the Hospital Quality Alliance (HQA). Besides other measures of hospital quality, the HC database provides the process of care measures and the Hospital Consumer Assessment of Healthcare Providers and Systems (HCAHPS) measures relevant for 2013 VBP score calculation. The process of care measures covers conditions like heart attack, heart failure, pneumonia, and surgical care improvement while the HCAHPS measures pertain to the patient experience of care. A detailed description of this data is provided in the CMS website:

<http://www.hospitalcompare.hhs.gov/staticpages/for-consumers/for-consumers.aspx> (Last accessed: December 15, 2011)

The Hospital Compare data was extracted from the Hospital Compare Access Database, which was downloaded from the following link: <http://www.medicare.gov/Download/DownloadDB.asp> (Last accessed: December 15, 2011). Once the database had been downloaded, the tables containing clinical process of care measures (CPC) data and HCAHPS data were read into SAS. A few transformations were needed to put it into the proper format, and the datasets were stripped down to only contain the 12 CPC and 8 HCAHPS measures currently being used to calculate VBP scores. Any CPC measures not meeting the minimum number of cases (10) were automatically given N/A scores. For each hospital in the database, CPC, HCAHPS and VBP scores were calculated using the formulas described in the Federal Register. Any hospital without valid CPC or HCAHPS data was given an N/A score for CPC or HCAHPS, respectively, and therefore, an N/A for their VBP score as well. Any hospital not meeting the following criteria was excluded from the final group: 1) A minimum of 100 HCAHPS survey responses, and 2) A minimum of 4 CPC measures with valid scores. Any hospital flagged as "Critical Access" in the Hospital Compare data was excluded from the final group as well. The final group of hospitals was linked to 2010 American Hospital Association Survey data (described below) by each hospital's provider number.

***American Hospital Association Annual Survey Database***

This is a survey of the US hospitals conducted by American Hospital Association (AHA) which collects data on hospital characteristics including demographics, organizational structure, facilities and services, utilization data, community benefit indicators, physician arrangements, managed care relationships, expenses, and staffing. The AHA registered hospital comprises about 98% of the survey universe that include about 6,500 hospitals in the US and the associated areas. Further details about this database including survey methodology can be obtained online from the following link:

<http://www.ahadata.com/ahadata/html/historymethodology.html> (Last accessed: December 15, 2011)

***Medicare Impact File***

The 2009 Medicare Impact File (MIF), prepared and maintained by CMS, provides relevant information for estimating payment impacts of various policy changes to the Medicare Inpatient Prospective Payment System (IPPS). Among other things, the MIF contains Disproportionate Share Index that measures the proportion of low-income patients served by the hospital and the ratio of interns and residents to the beds, which can be used as a proxy for the extent of teaching and education activities of a specific hospital. The following URL provides more information of the MIF:

<http://www.cms.gov/AcuteInpatientPPS/IPPS2009/itemdetail.asp?filterType=none&filterByDID=-99&sortByDID=2&sortOrder=ascending&itemID=CMS1220826&intNumPerPage=10> (Last accessed: December 15, 2011)

**List of Study Variables and their Respective Data Sources**

The following table provides the list of variables used in the study and their corresponding data sources.

Appendix Exhibit 2: Study Variables and Their Data Sources

| **Variables** | **Description** | **Data Source** | | |
| --- | --- | --- | --- | --- |
|  |  | **HC** | **AHA** | **MIF** |
| NUMMEASURES | Total number of clinical process of care measures reported by a hospital | * |  |  |
| CMIV25 | Case-mix Index |  |  | * |
| DSHPCT | Disproportionate Share Index as determined from cost report data & SSA data |  |  | * |
| MCR_PCT | Medicare days as percent of total inpatient days |  |  | * |
| MCD_PCT | Medicaid days as percent of total inpatient days |  |  | * |
| NURSE_STAFF | Ratio of total FTE for registered nurses to total inpatient days (=FTERN/IPDTOT) |  | * |  |
| FTERN | FTE for registered nurses |  | * |  |
| IPDTOT | Total inpatient days |  | * |  |
| TEACH_STATUS | Ratio of residents and interns to total beds (=FTRES/BDTOT) |  | * |  |
| FTRES | Full-time medical/dental residents |  | * |  |
| BDTOT | Total number of beds set up and staffed for use at the end of the reporting period |  | * |  |
| CNTROL (PROFIT STATUS) | 1 = For profit; 2 = Non-profit; 3 = Government-owned (non-federal) |  | * |  |
| Bedcat | 1 = "Beds 6-49"; 2 = "Beds 50-99"; 3 = "Beds 100-199"; 4 = "Beds 200-299"; 5 = "Beds 300-399"; 6 = "Beds 400-499"; 7 = "Beds 500 or MORE" |  |  | * |
| geographic region | 1 = "NEW ENGLAND"; 2 = "MID ATLANTIC"; 3 = "SOUTH ATLANTIC"; 4 = "EAST NORTH CENTRAL"; 5 = "EAST SOUTH CENTRAL"; 6 = "WEST NORTH CENTRAL"; 7 = "WEST SOUTH CENTRAL"; 8 = "MOUNTAIN"; 9 = "PACIFIC"; |  |  | * |
| URBAN | Whether urban hospital? |  |  | * |
| MNGT | Whether the hospital is contract managed? |  | * |  |
| MAPP1 | Whether the hospital is accredited by Joint Commission on Accreditation of Health Care Organizations (JCAHO) |  | * |  |
| MAPP8 | Member of Council of Teaching Hospital of the Association of American Medical Colleges (COTH) |  | * |  |
| NETWRK | Is the hospital a participant in a network |  | * |  |
| iphmohos | Health maintenance organization (HMO) hospital |  | * |  |
| obhos | Obstetrics care hospital |  | * |  |
| msichos | Medical/surgical intensive care hospital |  | * |  |
| cichos | Cardiac intensive care hospital |  | * |  |
| icfhos | Intermediate nursing care hospital |  | * |  |
| acuhos | Acute long term care hospital |  | * |  |
| mammshos | Breast cancer screening/mammograms hospital |  | * |  |
| aclabhos | Adult diagnostic catheterization hospital |  | * |  |
| adtchos | Adult cardiac surgery hospital |  | * |  |
| adtehos | Adult cardiac electrophysiology hospital |  | * |  |
| emdephos | Emergency Department hospital |  | * |  |
| endochos | Optical Colonoscopy hospital |  | * |  |
| gersvhos | Geriatric services hospital |  | * |  |
| occhshos | Occupational health services hospital |  | * |  |
| patedhos | Patient education center hospital |  | * |  |
| psyemhos | Psychiatric emergency services hospital |  | * |  |
| dradfhos | Diagnostic radioisotope facility hospital |  | * |  |
| mrihos | Magnetic resonance imaging (MRI) hospital |  | * |  |
| mscthos | Multislice spiral computed tomography < 64 slice hospital |  | * |  |
| wmgthos | Wound Management Services hospital |  | * |  |

Note:

1. HC=Hospital Compare; AHA=American Hospital Survey; MIF=Medicare Impact File

2. Variables in the lightly shaded rows are used to construct the preceding primary independent variable used in the analysis.
